# Predicting long-term sickness absence with employee questionnaires and administrative records: a prospective cohort study of hospital employees

**DOI:** 10.5271/sjweh.4124

**Published:** 2023-11-01

**Authors:** Solja T Nyberg, Marko Elovainio, Jaana Pentti, Philipp Frank, Jenni Ervasti, Mikko Härmä, Aki Koskinen, Laura Peutere, Annina Ropponen, Jussi Vahtera, Marianna Virtanen, Jaakko Airaksinen, G David Batty, Mika Kivimäki

**Affiliations:** 1 Clinicum, Faculty of Medicine, University of Helsinki, Helsinki, Finland.; 2 Finnish Institute of Occupational Health, Helsinki, Finland.; 3 Department of Psychology and Logopedics, Faculty of Medicine, University of Helsinki, Helsinki, Finland.; 4 Finnish Institute for Health and Welfare, Helsinki, Finland.; 5 Department of Public Health and Population Research Centre, University of Turku and Turku University Hospital, Turku, Finland.; 6 UCL Brain Sciences, University College London, London, UK.; 7 School of Educational Sciences and Psychology, University of Eastern Finland.; 8 Division of Insurance Medicine, Karolinska Institutet, Stockholm, Sweden.

**Keywords:** machine learning, risk prediction, survey data

## Abstract

**Objective:**

This study aimed to compare the utility of risk estimation derived from questionnaires and administrative records in predicting long-term sickness absence among shift workers.

**Methods:**

This prospective cohort study comprised 3197 shift-working hospital employees (mean age 44.5 years, 88.0% women) who responded to a brief 8-item questionnaire on work disability risk factors and were linked to 28 variables on their working hour and workplace characteristics obtained from administrative registries at study baseline. The primary outcome was the first sickness absence lasting ≥90 days during a 4-year follow-up.

**Results:**

The C-index of 0.73 [95% confidence interval (CI) 0.70–0.77] for a questionnaire-only based prediction model, 0.71 (95% CI 0.67–0.75) for an administrative records-only model, and 0.79 (95% CI 0.76–0.82) for a model combining variables from both data sources indicated good discriminatory ability. For a 5%-estimated risk as a threshold for positive test results, the detection rates were 76%, 74%, and 75% and the false positive rates were 40%, 45% and 34% for the three models. For a 20%-risk threshold, the corresponding detection rates were 14%, 8%, and 27% and the false positive rates were 2%, 2%, and 4%. To detect one true positive case with these models, the number of false positive cases accompanied varied between 7 and 10 using the 5%-estimated risk, and between 2 and 3 using the 20%-estimated risk cut-off. The pattern of results was similar using 30-day sickness absence as the outcome.

**Conclusions:**

The best predictive performance was reached with a model including both questionnaire responses and administrative records. Prediction was almost as accurate with models using only variables from one of these data sources. Further research is needed to examine the generalizability of these findings.

Long-term sickness absence is an indicator of poor employee health, lost work contributions, and increased risk of early labor market exit and premature mortality (1–4). To reduce sickness absence, interventions have targeted employees at the highest risk (5, 6). Several prediction algorithms have been developed to identify these individuals. Some models have been based on data collected via questionnaire survey (7–9), others on routine administrative register data (10) and still others using items from both sources (11–16). Typical predictors include sociodemographic characteristics, such as sex, age, education, and socio-economic status; health-related factors, such as self-rated health and previous sickness absences; and, occasionally, some work-related psychosocial indices such as work demands (7–16).

Each source of data has its strengths and weaknesses. Employee questionnaires enable the collection of information on work-, behavior- and health-related predictors of sickness absence, but unless it is a short online survey, questionnaire implementation often requires substantial resources to attain a high response (17, 18). Further potential drawbacks in self-administered questionnaires are recall bias and missing data due to low response rates, which can cause error in risk modelling (19). The benefits of using administrative data include automatic data collection, little or no missing data, lower costs (20, 21), and, perhaps most importantly, zero burden to the study participant. These data typically include information on working hours and work unit characteristics, such as turnover rates and the demographic structure of the staff, but lack important predictors of sickness absence, such as self-reported behavioral, health-related and psychosocial factors (22–25). While occupational health services can handle sensitive self-report data needed for survey responses, employers have full access to administrative data.

In this study, we compared algorithms that estimate an individual’s risk of long-term sickness absence based on questionnaire responses to the Finnish Institute of Occupational Health (FIOH) risk prediction score (26), administrative records of workplace and working hour characteristics (27), and a combination of both data sources. The FIOH score comprises just eight concise questions, requiring approximately two minutes to complete, thereby minimizing participant burden. When predicting work disability, these eight questionnaire variables have accounted for over 99% of the variance in a questionnaire that included 82 sociodemographic, health status, lifestyle, and work-related psychosocial variables (26). Our administrative records encompass 28 variables related to workplace characteristics and working hours, which have previously been validated (27). Our study focused on shift workers due to the routine collection of working hour characteristic information for this employee group.

## Methods

### Study population

Data were from one of the participating hospital districts of the Finnish Public Sector study, which is a prospective cohort study of public sector employees from 10 municipalities and 21 hospitals in the same geographical areas in Finland (28). The subset of shift workers was chosen because study participants had data from both a questionnaire survey on predictors of work disability and administrative data on working hours and workplace characteristics. We included participants who responded to the survey in 2015, were linked to administrative data in the same year, and had a minimum follow-up period of 150 days for sickness absence after 2015. All participants were eligible for sickness absence. Approval was obtained from the ethics committee of the Helsinki-Uusimaa Hospital District Ethics Committee (HUS/1210/2016).

### Potential predictors of long-term sickness absence

Participant-completed questionnaire survey items of the validated FIOH risk calculator for the prediction of work disability in the general working population include: age group (<35; 35–39; 40–44; 45–49; 50–54; ≥55 years), body mass index (BMI) (<18.5, 18.5–<25; 25–<30; ≥30 kg/m^2^), socioeconomic status (SES) (low; intermediate; high), smoking (yes or no), number of chronic diseases (0; 1; 2; ≥3), self-rated health (good; fairly good; moderate; fairly poor; poor), difficulty falling asleep (not at all; 1–3 times/month; about once a week; 2–4 nights/week; 5–6 nights per week; every night), and the number of sickness absence episodes >9 days during one year before baseline (0; 1; 2; ≥3) (26).

**Figure 1:**
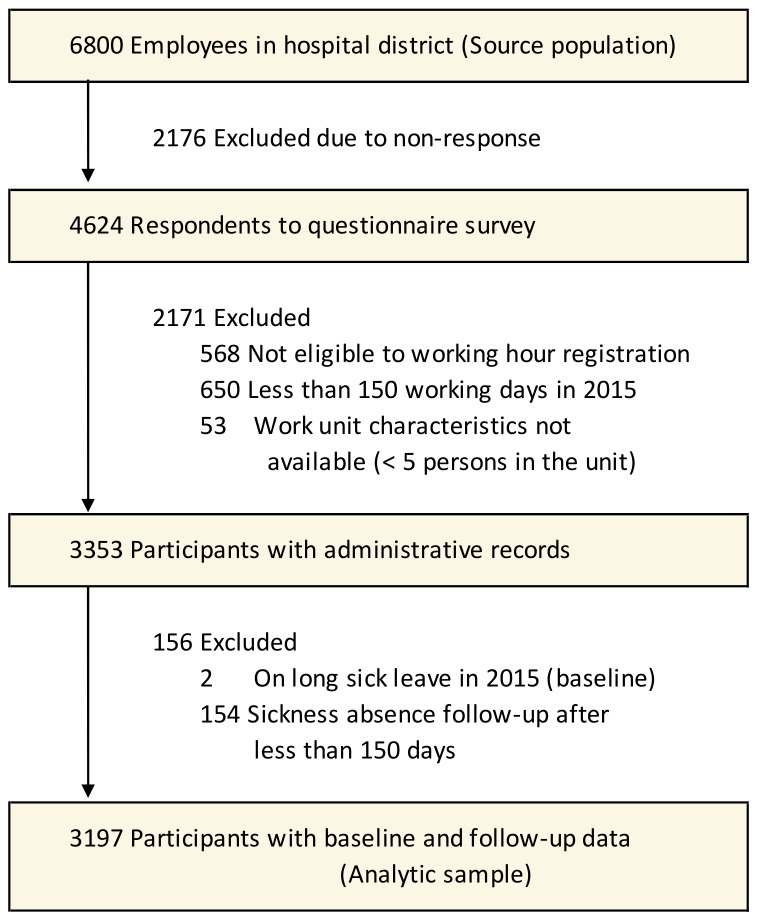
Flow chart for sample selection.

Administrative records comprised individual-level data on 18 characteristics of working hours (27). The times of daily working hours and the reasons for an absence (day off, sick leave, maternity leave, physician’s on-call duties, annual leave etc) were retrieved from a shift scheduling software Titania® (CGI Finland) and annual working hours were calculated. The variables from these records were calculated for those who had ≥150 working days in 2015 and are presented in detail in table 1. Further administrative records for the year 2015 were retrieved from human resources management registries of the workplaces. Using these records, we defined ten characteristics of the participant’s working unit: the number of employees, mean age of employees, proportion of employees aged ≤30, proportion of employees aged ≥60, proportion of non-permanent employees, proportion of nurses, proportion of employees with low SES, turnover (last 2 years; between autumn 2013 and autumn 2015), turnover (last 4 years; between autumn 2011 and autumn 2015). Low SES referred to ‘service workers’, ‘process workers’ and ‘other/elementary occupations’, according to the International Standard Classification of Occupations (29). In line with data protection regulations, we excluded data from small work units (N<5) from the analysis and computed these characteristics only for work units comprising ≥5 employees (figure 1).

**Table 1:** Baseline characteristics. [SD=standard deviation; SES=socioeconomic status.]

Baseline characteristic		All (N=3197)			Sickness absence case (N=190)			Non-case (N=3007)		P-value
		N (%)	Mean (SD)		N (%)	Mean (SD)		N (%)	Mean (SD)	
**Questionnaire**										
	Sex									0.119
	Women	2814 (88.0)			174 (91.6)			2640 (87.8)		
	Men	383 (12.0)			16 (8.4)			367 (12.2)		
Age			44.5 (11.0)			49.3 (9.3)			44.2 (11.0)	<0.001
Socioeconomic status										<0.001
	Low	501 (15.7)			50 (26.3)			451 (15.0)		
	Intermediate	2156 (67.4)			118 (62.1)			2038 (67.8)		
	High	540 (16.9)			22 (11.6)			518 (17.2)		
Self-rated health										<0.001
	Good (1, 2)	2685 (84.0)			123 (64.7)			2562 (85.2)		
	Moderate or poor (3–5)	512 (16.0)			67 (35.3)			445 (14.8)		
Body mass index (kg/m^2^)										0.007
	<25	1542 (48.2)			73 (38.4)			1469 (48.9)		
	25–<30	1154 (36.1)			75 (39.5)			1079 (35.9)		
	≥30	501 (15.7)			42 (22.1)			459 (15.3)		
Smoking										<0.001
	Yes	356 (11.1)			38 (20.0)			318 (10.6)		
	No	2841 (88.9)			152 (80.0)			2689 (89.4)		
No. of chronic diseases										0.002
	0	2071 (64.8)			102 (53.7)			1969 (65.5)		
	1	918 (28.7)			66 (34.7)			852 (28.3)		
	2	180 (5.6)			}22 (11.6) ^a^			162 (5.4)		
	≥3	28 (0.9)						24 (0.8)		
Trouble falling asleep										0.074
	Max 3 times / month	2402 (75.1)			130 (68.4)			2272 (75.6)		
	1–4 times / week	693 (21.7)			51 (26.8)			642 (21.4)		
	5 times / week or more	102 (3.2)			9 (4.7)			93 (3.1)		
No. of sickness absences during the previous year										<0.001
	0	2722 (85.1)			132 (69.5)			2590 (86.1)		
	1	388 (12.1)			39 (20.5)			349 (11.6)		
	2	72 (2.3)			}19 (10.0) ^a^			56 (1.9)		
	≥3	15 (0.5)						12 (0.4)		
**Administrative records**										
Working hour characteristics (proportion of)										
	Long (>40 hour) working weeks		34.9 (2.8)			34.0 (4.3)			34.9 (2.7)	<0.001
	Long (>48 hour) working weeks		19.7 (15.3)			17.9 (15.9)			19.8 (15.3)	0.089
	Long shifts		3.9 (13.2)			3.8 (13.7)			3.9 (13.2)	0.969
	Long night shifts		1.5 (7.1)			1.7 (7.1)			1.5 (7.1)	0.778
	Early morning shifts		0.1 (2.5)			0.6 (7.3)			0.1 (1.9)	0.007
	Morning shifts		70.7 (27.4)			68.0 (28.1)			70.9 (27.3)	0.162
	Day shifts		4.2 (11.3)			6.6 (16.1)			4.0 (11.0)	0.003
	Evening shifts		16.4 (16.8)			17.4 (17.5)			16.4 (16.8)	0.397
	Night shifts		8.6 (15.0)			7.4 (14.0)			8.6 (15.0)	0.260
	Non-day shifts		25.1 (26.9)			25.4 (26.5)			25.1 (26.9)	0.877
	Long spells of work shifts		1.9 (3.8)			1.4 (2.9)			1.9 (3.9)	0.083
	Short shift intervals		8.5 (11.3)			8.2 (12.0)			8.5 (11.3)	0.718
	Annual leave days		12.3 (4.1)			12.7 (4.2)			12.3 (4.1)	0.281
	Week-end work		22.8 (23.6)			22.5 (23.5)			22.8 (23.6)	0.873
	Single free days		10.1 (9.2)			10.7 (10.0)			10.1 (9.2)	0.411
	Realised shift plans		93.6 (7.6)			93.9 (7.1)			93.6 (7.6)	0.685
	Use of shift wishes		9.5 (15.5)			8.0 (13.9)			9.6 (15.6)	0.175
Work unit characteristics										
	Number of staff		59.0 (43.7)			53.4 (38.5)			59.4 (44.0)	0.070
	Mean age of staff		44.3 (3.7)			45.2 (4.1)			44.2 (3.7)	<0.001
	Proportion of employees aged ≤30		14.0 (8.9)			12.3 (8.8)			14.1 (8.9)	0.008
	Proportion of employees aged ≥60		9.3 (6.2)			11.2 (7.9)			9.2 (6.1)	<0.001
	Proportion of non-permanent staff		20.3 (9.6)			18.9 (9.1)			20.4 (9.7)	0.030
	Proportion of nurses		45.4 (33.3)			41.5 (34.6)			45.7 (33.2)	0.092
	Proportion of staff with low SES		15.9 (25.2)			25.9 (34.5)			15.2 (24.3)	<0.001
	Turnover (last 2 years)		20.3 (11.1)			19.4 (10.0)			20.4 (11.1)	0.258
	Turnover (last 4 years)		30.9 (14.4)			30.4 (14.7)			31.0 (14.4)	0.566
	Rate of long sickness absence in the unit		0.6 (0.3)			0.7 (0.3)			0.6 (0.3)	<0.001

^a^ Results for groups with N ≤5 were merged into other groups for data protection purposes.

A complete list and categorization of the potential predictors is provided in table 1 and the supplementary material,www.sjweh.fi/article/4124, table S1.

### Ascertainment of long-term sickness absence during the follow-up

In Finland, sickness absence periods lasting >9 days and with medical certification are recorded in the Social Insurance Institution. Employees receive compensation based on their salary during their sickness absence for up to 300 weekdays. If a sickness absence lasts ≥90 days, to be entitled for compensation, the employee needs to provide the Finnish Social Insurance Institution with a detailed certificate from an occupational physician about his/her inability to work (30).

We used administrative data to ascertain sickness absences at follow-up, including long-term sickness absence records between 1 January 2016 and 31 December 2019. As in previous studies, outcome was the first ≥90-day sickness absence (14). The first >30-day sickness absence was our secondary outcome.

### Statistical analyses

There were no missing data on administrative records at baseline or sickness absence at follow-up. The amount of missing values for the 8 questionnaire items was small: 3% for BMI (N=101) and the number of chronic diseases (N=92), <1% for smoking (N=18), self-rated health (N=12) and trouble falling asleep (N=25). As these questionnaire items were categorical, we imputed missing values using the mode value from the entire study population. For comparison, we repeated the main analyses in the 2981 participants with no missing data (93.2% of the total sample, the complete case analysis).

Analysis of long-term sickness absence prediction included the following three steps. First, we examined separately three predictive models including (i) all 8 pre-defined questionnaire-based items (ii), all 28 administrative data-based items, and (iii) all 36 items from both sources. We obtained item coefficients from logistic regression and constructed a risk score for each participant using the predicted risk from the model. We compared the three prediction models to determine which data source or sources provided the best prediction for long-term sickness absence as indicated by C-index and other prediction metrics. C-index gives the probability that a randomly selected individual who experienced the outcome during the follow-up, had a higher risk score than a randomly selected individual who did not experience the outcome. The C-index ranges from 0.5 (no predictive ability) to 1 (maximum predictive ability). C-index under 0.7 represents poor, 0.7–0.8 good, and >0.8 strong discrimination ability (31). As recently suggested (32, 33), we additionally examined detection rate, false positive rate, and the ratio of true to false positives using different thresholds for stratifying the population into high risk (test positive) versus low risk (test negative). Formulas for these statistics are as follows:

*False positive rate* (the proportion of test positive cases who did not experience work disability) = b/(b+d)

*Detection rate* (the proportion of work disability cases who were test positive) = a/(a+c)

*Ratio of true to false positives* = 1: (b/a), where a, b, c and d represent different combinations of risk scores and work disability as defined below:

**Table:** 

	Work disability during the follow-up	
Risk score	Yes	No
Test positive	a	b
Test negative	c	d

The alternative thresholds for a test positive result were set at 5%, 10%, 15% and 20% of model predicted risk.

To evaluate whether the best model was associated with the outcome at each year of the follow-up, we computed cumulative incidence of long-term sickness absence by follow-up year (1–4) and high versus low risk score. Relative importance of each predictor was illustrated using -10log(P-value).

Second, to examine the robustness of evidence, we investigated whether the same source or sources as in the main analysis provided the best prediction also after reducing the number of predictors. For dimension reduction (feature selection), we used several alternative approaches, including traditional stepwise logistic regression modelling and five different methods of machine learning with cross-validation. We used stepwise logistic regression with forward and backward variable selection based on Akaike Information Criteria (AIC). Machine learning models included: (i) LASSO (least absolute shrinkage and selection operator) regression, which is a regression analysis method that performs feature selection and regularization. This method performs L1 regularization. The results were based on k-fold cross-validation with k=10 (34); (ii) ridge regression, which is a model tuning method that is used to analyze any data that suffers from multicollinearity. This method uses L2 regularization. The results were based on k-fold cross-validation with k=10 (34); (iii) elastic net, which is a regularized regression method that linearly combines the L1 and L2 penalties of the Lasso and Ridge methods – the results were based on repeated k-fold cross-validation with k=10 (34); (iv) genetic algorithm (GA), which is a stochastic search algorithm inspired by the basic principles of biological evolution and natural selection – the results were based on k-fold cross-validation with k=10 (35); and (v) Boruta, which is a random forest-based feature selection algorithm for finding relevant variables (36).

Instead of generating new risk scores, the objective of these analyses was to determine whether variable selection would favor questionnaire-based or register-based predictors or encompass predictors from both data sources.

Third, we repeated the main analysis as described in step 1 using the first >30-day sickness absence (instead of the first >90-day sickness absence) as the outcome.

All analyses were performed using SAS 9.4 (SAS Institute, Cary, NC, USA) and R 4.2.2, including packages Boruta (36), glmnet (34), caret (37), and odds.n.ends (38).

## Results

The baseline population comprised 6800 participants (figure 1). Of these, 4624 responded to the questionnaire (response rate 68%) and 3990 were also eligible to working hour registration and had work unit data available. Of them, in order to capture sickness absence incidence, we excluded 53 participants who were on long-term sickness absence at baseline and 740 with missing data on administrative predictors or long-term sickness absence at follow-up. The analytical sample included 3197 individuals. They did not differ from the baseline population in terms of sex (proportion of women 88% in the analytic sample and 84% in the baseline population) or age (mean 44.5 vs 44.8 years). Table 1 shows the baseline characteristics of the study participants by sickness absence at follow-up.

**Figure 2:**
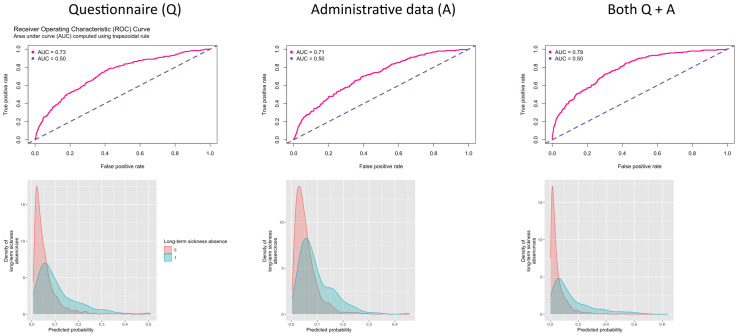
Area Under the Curve (AUC) (upper panel) and distribution of risk scores by long-term sickness absence at follow-up (lower panel).

During a 4-year follow-up (mean 3.5, SD 0.9, years), 190 (5.9%) participants were denoted as taking long-term sickness absence. Figure 2 shows the AUC curves and the density functions by long-term sickness absence for prediction models using all the 8 questionnaire items, all the 28 administrative variables and both of these sets of variables. The C-index was 0.73 (95% CI 0.70–0.77) for the questionnaire-based predictor, 0.71 (95% CI 0.67–0.75) for administrative data and 0.79 (95% CI 0.76–0.82) for a model including all 36 questionnaire and administrative items, the last model providing a slightly better prediction than the first two models. Similar results were obtained for participants with no missing data (complete cases analysis) with C-index being 0.79 (95% CI 0.76–0.82) for a model including all 36 questionnaire and administrative items. The distributions of the three prediction models were highly overlapping between the long-term sickness absence cases and non-cases.

All the six approaches for feature selection included both questionnaire items and administrative variables, confirming the findings based on all predictors (supplementary table S2). C-indices varied between 0.72 and 0.77, indicating that the predictive models based on reduced number of predictors did not achieve the level of performance of the model which included all 36 questionnaire and administrative variables.

**Figure 3:**
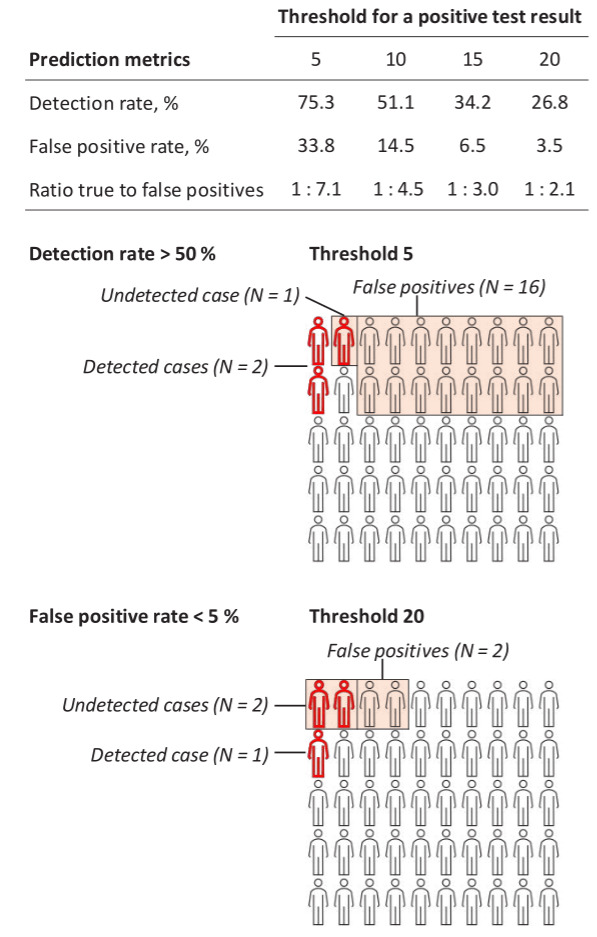
Capacity of the 36-item risk score to estimate risk of long-term sickness absence with two illustrations.

Figure 3 shows detection rate, false positive rate, and the ratio of true to false positives for different cut-off points to define a positive test result for the 36-variable model. For cut-off points of 5% and 20% risks for a positive test result, the detection rates were 75.3% and 26.8%, respectively. The corresponding false positive rates were 33.8% and 3.5%. For one true positive case, the expected number of false positive cases was 7.1 for the 5%-risk cut-off and 2.1 for the 20%-risk cut-off.

Figure 3 also presents an illustration of the number of test positives divided into true and false positives and the number of incident cases missed by the test, within a group of 50 persons in which three would have long-term sickness absence at follow-up. Using the 5% risk threshold for a positive test, the model would detect 2 sickness absence cases, miss 1 case and assign 16 non-cases falsely as test positives. With a 20%-risk threshold, the model would detect 1 case, miss 2 cases and assign 2 non-cases as test positives. To detect at least half of the cases (N=2), the threshold for positive test should be set an estimated 10%-risk. With this cut-off, one case was detected, 2 missed and 40 were false positives.

**Figure 4:**
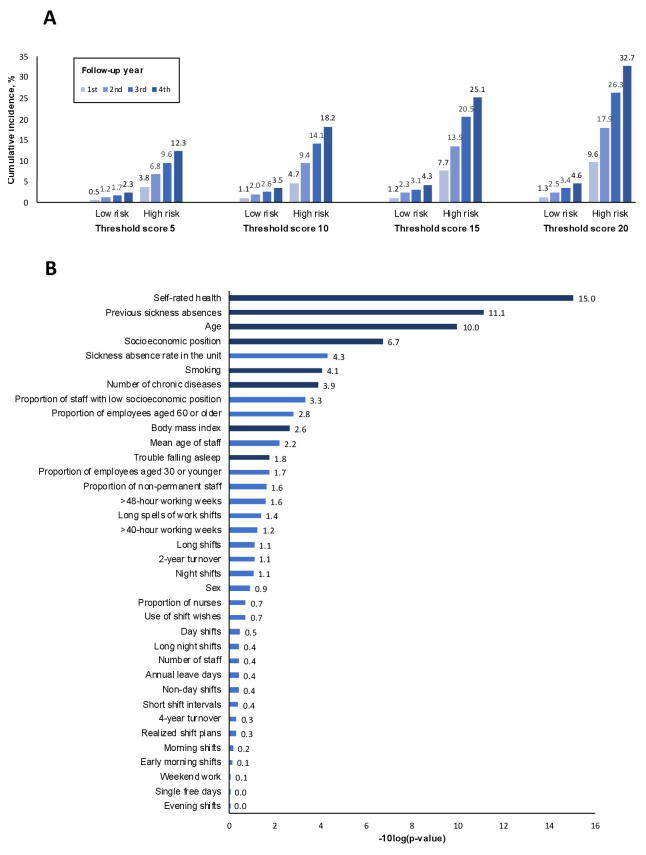
Cumulative incidence of long-term sickness absence by follow-up year and level of 36-item risk score (A) and relative importance of the items of the score* (B).*Dark blue=questionnaire item; light blue=administrative data.

Figure 4, Part A, illustrates the gradual increase in the separation of cumulative incidence of sickness absence between individuals with high and low risk scores across all years of follow-up, regardless of the chosen cut-off for defining a positive test result. In Part B, the relative importance of predictors is demonstrated in terms of -10log(p); self-rated health, previous sickness absence, socioeconomic status, and the sickness absence rate in the participant’s working unit were the strongest predictors.

In supplementary tables S3-S5, we report these values for the models including all items from either questionnaire or administrative data only, along with sensitivity, specificity, positive predictive values and negative predictive values for all these models. With an estimated 5%-risk threshold for a test positive result, a prediction model based on questionnaire items only has a detection rate of 75.8%, false positive rate of 33.8 and the ratio true-to-false positives of 1:8.4. With the threshold raised at estimated 20% risk, the corresponding figures are 14.2%, 2.4% and 1:2.7. For a prediction model using only administrative data, detection rate is 73.7%, false positive rate is 45.2% and the ratio true-to-false positives is 1:9.7 for a 5% risk threshold. The corresponding metrics are 8.4%, 1.7% and 1:3.2 for an estimated 20% risk threshold.

Repeating the analyses with our secondary outcome (>30-day sickness absence, incidence 21.5%, 687 incident cases) replicated the results of the primary outcome (supplementary tables S6-S7, figures S1-S2). A model incorporating both questionnaire items and administrative variables yielded a slightly enhanced prediction (C-index 0.71) compared to models relying solely on one of these data sources (C-index 0.67 for questionnaire items only and 0.65 for administrative variables only). All the six approaches for feature selection included both questionnaire items and administrative variables.

## Discussion

Using data from a prospective cohort study of 3197 hospital employees, we compared the predictive performance for long term sickness absence (>90 days) between models including items from either questionnaire responses or administrative records, or both. C-indices between 0.7 and 0.8 suggested that discriminatory ability of all the models was good. The best predictive performance for long-term sickness absence was found for a model including both questionnaire responses and routine administrative records. However, detection rates, false positive rates, and the ratio true-to-false positives suggest that in practice prediction was almost as accurate with models using only variables from one of these data sources.

The C-index for both single source models predicting long-term sickness absence exceeded 0.70 although the highest C-index (0.79) was achieved with a model including all the 36 potential predictors from both sources. We used several machine learning approaches to identify a model with high discriminative ability with less items, but none of the C-indices (variation between 0.72 and 0.77) for these models reached the predictive performance of the model with all 36 potential predictors. Additionally, all these models included both administrative and questionnaire variables, with the number of predictor items varying between 9 and 27 depending on the method of feature selection.

The model with all 36 predictor items may have clinical value in risk stratification. For dichotomized tests with cut-off points for a positive test result varying between 5% (a model for a high detection rate) and 20% (a model for low false positive rate) estimated risk, the detection rate varied between 75.3% and 26.8%, respectively. The corresponding false positive rates were 33.8% and 3.5% and the ratios true-to-false positives 1.0 to 7.1 and 2.1.

### Comparison with other studies

Several studies on sickness absence prediction have been conducted, with risk prediction models typically showing varying predictive capacities. C-indices or AUC for a prediction model for sickness absence have been as high as 0.80 (16), while some studies have presented models with good discrimination (C-index or AUC less than 0.80 but exceeding 0.7) (7, 10, 14) or modest discrimination values (C-index or AUC<0.7) (11, 13). However, we are not aware of previous studies comparing the predictive performance between models using different combinations of questionnaire and administrative data. Furthermore, few studies have reported detection and false positive rates and the ratio true-to-false positives of the models, although these metrics are important to assess the clinical value of prediction algorithms.

In recent years, an array of clinical tools for predicting the risk of specific chronic diseases have been established, some of which are already in use in clinical practice. The predictive capacities of these models are comparable with those for long-term sickness absence. For example, the Pooled Cohort Risk Equations for predicting the 10-year risk of cardiovascular disease events yielded a C-index of 0.72 (39), whereas the C-index of the FINDRISK model for predicting type 2 diabetes have varied between 0.74 and 0.77 (40, 41), and that for the QRISK3 model for the prediction of cardiovascular disease risk between 0.70 and 0.91 (42).

We have previously validated a risk score for work ability, and further tested its predictive performance among employees with chronic conditions (26, 43). Among the different disease groups, with an estimated 30% risk cut-off, the detection rates varied between 42% and 80%, and false positive rates between 10% and 46%, depending on the disease group under investigation. For a lower cut-off (5%) for test positive result, the ranges of the corresponding indicators were 92–99% and 54–94%, respectively.

One previous study of prediction models for workplace bullying was conducted using both survey responses and administrative records (44). The predictive performance of the models obtained was modest, with C-indices <0.7. For a low (5%) cut-off for a positive test result of being bullied, the authors found relatively high detection rates (79–84%) although this was accompanied by high false positive rates (56–71%). With a 15% cut-off, the false positive rates were low (1–5%), but the detection rates dropped to 3–17% (44). These results are comparable to those from our study, in which a low cut-off point (5%) yielded a detection rate of 75%, whereas a 20% cut-off point reduced the detection rate to 27%. The corresponding false positive rates were 33.8% and 3.5%, respectively.

### Strengths and limitations of this study

Strengths of this study include the use of informative metrics to evaluate the clinical value of the prediction algorithms, the assessment of predictors across two sources of data and ascertainment of long-term sickness absence through administrative records covering all employees. In addition to C-statistics, we evaluated clinical value with detection and false positive rates and the ratio true to false positives. Few previous studies on prediction models for sickness absence have reported these metrics (45).

There are, however, some important limitations. No independent study population was available for external validation or examination of the reproducibility of our findings. As long-term sickness absence is defined as temporary work disability, receipt of a sickness absence is associated with medical and non-medical factors, such as sickness absence regulations, the work environment, the nature of the job, and the extent to which a workplace is prepared to accommodate the disability. In addition, our target population was limited to shift-working hospital employees and included more women than men. Thus, the findings of this study may not be generalizable to other settings and should be validated in other countries and study populations.

### Concluding remarks

In conclusion, when evaluating the utility of alternative prediction algorithms for long-term sickness absence among shift workers, we compared models based on employee questionnaires and administrative records. Our findings revealed that neither source outperformed the other; instead, the highest predictive accuracy was achieved when both data sources were combined.

In practice, combining responses from two different sources may not always be straightforward to implement because occupational health services do not usually have access to administrative data and the employers mainly do not have the authority to handle sensitive self-report data such as chronic conditions and self-rated health needed for survey responses. According to our results, both sources alone performed reasonably well separately and could therefore be recommended to be used for risk prediction.

Our findings can guide the implementation of targeted interventions, as decisions concerning target groups often necessitate the use of dichotomized risk models to identify individuals at risk – in this context, those with a high probability of experiencing long-term sickness absence. For meaningful resource allocation, the performance of the prediction models is important, and the intervention’s characteristics should be factored into the threshold chosen for a positive test result. When implementing cost-effective interventions with no negative side effects, it is preferable to set low risk thresholds for a positive test result. This approach enhances detection rates while simultaneously accommodating relatively high false-positive rates. However, in cases where the intervention is costly or has an unknown safety profile with potential adverse effects, minimizing false-positive rates is favored to mitigate harms. In the present study, models allowing a correct prediction for the majority of the study population had relatively high false positive rates suggesting that these predictive algorithms are more suitable for the implementation of inexpensive interventions with no negative side effects.

### Ethics approval and consent to participate

Ethical approval was obtained from the ethics committee of the Helsinki-Uusimaa Hospital District Ethics Committee (HUS/1210/2016). Informed consent was obtained from all individual participants included in the study.

## Supplementary material

Supplementary material

## Data Availability

The statistical syntax used for the analysis of the present study is available in the supplementary material on page 12. Requests for pseudonymised data should be directed to Dr. Jenni Ervasti (jenni.ervasti@ttl.fi) and Prof. Mikko Härmä (mikko.harma@ttl.fi) (data owner). According to the data sharing agreement between FIOH and the organisations from which the data were collected, access to and analysis of the data is limited to employees and visiting investigators at FIOH.
