# Supplementary material for: Predicting long-term sickness absence with employee questionnaires and administrative records: a prospective cohort study of hospital employees
Source: Scand J Work Environ Health. 2023 Oct 31;49(8):610–20. doi: 10.5271/sjweh.4124 (PMC10882516; doi:10.5271/sjweh.4124)
Supplement: Supplementary material [file SJWEH-49-610-S001.pdf]

Predicting long-term sickness absence with employee questionnaires and administrative records: a prospective cohort study of hospital employees[1]

by Solja T Nyberg, PhD,[2] Marko Elovainio, PhD, Jaana Pentti, MSc, Philipp Frank, PhD, Jenni Ervasti, PhD, Mikko Härmä, MD, Aki Koskinen, MSc, Laura Peutere, PhD, Annina Ropponen, PhD, Jussi Vahtera, MD, PhD, Marianna Virtanen, PhD, Jaakko Airaksinen, PhD, G David Batty, DSc, Mika Kivimäki, FMedSci

1. Supplementary material
2. Correspondence to: Dr. Solja T Nyberg, University of Helsinki, Clinicum, Faculty of Medicine, Tukholmankatu 8B, FI-00014 Helsingin yliopisto, Finland. [E-mail: solja.nyberg@helsinki.fi]

List of Contents

Supplementary Table S1. List of potential predictors 2

Supplementary Table S2. Variables selected by six approaches for feature selection 4

Supplementary Table S3. Predictive performance of a 36-item model based on questionnaire and administrative data using five alternative thresholds for a positive test result 5

Supplementary Table S4. Predictive performance for models based on questionnaire data only or administrative data only 6

Supplementary Table S5. Sensitivity, specificity and predictive value for models based on questionnaire only, administrative data only or predictors from both sources 7

Supplementary Figure S1. Area Under Curve (AUC) for a 36-item model in relation to primary outcome (>90-day sickness absence) and secondary outcomes (>30-day sickness absence) 8

Supplementary Table S6. Predictive performance of a 36-item model in relation to primary outcome (>90-day sickness absence) and secondary outcomes (>30-day sickness absence) 9

Supplementary Figure S2. Illustration of the capacity of the 36-item risk score to estimate risk of long-term sickness absence for primary and secondary outcomes 10

Supplementary Table S7. Variables selected by six approaches for feature selection (secondary outcome) 11

Supplementary Methods: 12

Statistical code 12

Supplementary Table S8. STROBE Checklist 15

Supplementary Table S1. List of potential predictors

ADMINISTRATIVE DATA (WORKING HOUR CHARACTERISTICS BASED ON DAILY WORKING HOURS)

Length of working hours

Working hours included the average weekly (from Monday 00:00 to Sunday 24:00) working hours during the year. Calendar weeks without any work, that is on paid or non-paid leave, were excluded

Long (>40 hour) working weeks (%)

The proportion of calendar weeks of >40 weekly hours of all calendar weeks with work during the year

Long (>48 hour) working weeks (%)

The proportion of calendar weeks of >48 weekly hours of all calendar weeks with work during the year

Long shifts (%)

The proportion of ≥12 hours shifts/all shifts during the year

Long night shifts (%)

The proportion of ≥12-hour night shifts/all shifts during the year

Time of the day

Early morning shifts

The proportion (%) of early morning shifts /all shifts during the year

Morning shifts

The proportion (%) of morning shifts /all shifts during the year

Day shifts

The proportion (%) of day shifts /all shifts during the year

Evening shifts

The proportion (%) of evening shifts /all shifts during the year

Night shifts

The proportion (%) of night shifts /all shifts during the year

Non-day shifts

The proportion (%) of non-day shifts (=early morning, evening, or night shifts)/all shifts during the year

Shift intensity

Long spells of work shifts

Proportion (%) of >6 consecutive daily work shifts (without free days)/ all spells of consecutive daily work shifts)

Short shift intervals

The proportion (%) of shift intervals of ≤11 hours during the year/ all shift intervals

Social aspects of working hours

Annual leave days

Proportion (%) of annual leave days/ annual contract day

Week-end work

Proportion (%) of Saturday and/or Sunday work/ all week-ends

Single free days

Proportion (%) of single free days/all free days

Realized shift plans

Proportion (%) of all realized annual shifts /all planned shifts (based on the comparison of the planned and finalized shift plans)

Use of shift wishes

Proportion (%) of wished shifts/all shifts

ADMINISTRATIVE DATA: WORKPLACE UNIT -BASED AGGREGATED VARIABLES

Number of staff
Mean age of staff
Proportion of employees aged 30 or younger (%)
Proportion of employees aged 60 or older (%)
Proportion of non-permanent staff (%)
Proportion of nurses (%)
Proportion of staff with low employment grade (ISCO 5-9, %)
Turnover (last 2 years) (%)
Turnover (last 4 years) (%)
Rate of long sickness absence in the unit (year 2015)

QUESTIONNAIRE DATA

Age
BMI
    Height in cm
    Weight in kg
SES
    Participants' job title, categorised based on the International Standard Classification of Occupations (ISCO) as follows:
    high: 1 = manager/higher official, 2 = senior specialist
    intermediate: 3 = specialist, 4 = office worker
    low: 5 = service worker, 6 = process worker, 7 = other/elementary occupations.
Smoking
    Do you smoke or have you smoked regularly (every day or almost every day)? (Yes / No)
    Do you still smoke regularly? (Yes / No)
Number of chronic diseases (of the following diseases [0, 1, 2, 3+])
    Bronchial asthma
    Myocardial infarction
    Angina pectoris
    Cerebrovascular diseases
    Migraine
    Depression
    Diabetes
Self-rated health
    How is your state of health? (Good, rather good, moderate, rather poor, poor)
Trouble falling asleep
    How many times in the past 4 weeks have you had trouble falling asleep (not at all, 1-3 times per month, approximately once a week, 2-4 times per week, 5-6 times per week, every night)
Number of sickness absences in previous year (0, 1, 2, 3+)

Supplementary Table S2. Variables selected by six approaches for feature selection

**Predictor**	**Stepwise**	**Boruta**	**Lasso**	**Ridge**	**GA**	**Elastic net**
**Corresponding AUC**	**0.76**	**0.77**	**0.77**	**0.75**	**0.72**	**0.77**
**Administrative data**						
Sex			●		●	
Long (>40 hour) working weeks					●	
Long (>48 hour) working weeks		●	●		●	
Long shifts		●				
Long night shifts		●				
Early morning shifts						
Morning shifts		●			●	
Day shifts						
Evening shifts		●			●	
Night shifts		●				
Non-day shifts		●	●		●	
Long spells of work shifts		●	●		●	
Short shift intervals		●			●	
Annual leave days	●	●	●			●
Week-end work		●			●	
Single free days	●	●	●			●
Realized shift plans		●	●			●
Use of shift wishes		●				
Number of staff	●	●	●			●
Mean age of staff		●			●	
Proportion of employees aged 30 or younger		●			●	
Proportion of employees aged 60 or older	●		●	●	●	●
Proportion of non-permanent staff		●				
Proportion of nurses		●				
Low SES		●	●	●		●
Turnover (last 2 years)		●	●			
Turnover (last 4 years)		●			●	
Rate of long sickness absence in the unit	●	●	●	●	●	●
**Questionnaire data**						
Age group	●	●	●	●		●
Body mass index (kg/m2) category			●		●	●
Socioeconomic status				●	●	
Smoking	●	●	●	●		●
No. of chronic diseases			●	●	●	●
Self-rated health	●	●	●	●	●	●
Trouble falling asleep			●	●	●	●
No. of sickness absences during the previous year	●	●	●	●		●

Supplementary Table S3. Predictive performance of a 36-item model based on questionnaire and administrative data using five alternative thresholds for a positive test result

Prediction metrics					
	Outcome: Sickness absence > 90 days				
Threshold for a positive test result	1	5	10	15	20
Detection rate, %	99.5	75.3	51.1	34.2	26.8
False positive rate, %	86.7	33.8	14.5	6.5	3.5
Ratio true to false positives	1 : 13.3	1 : 7.1	1 : 4.5	1 : 3.0	1 : 2.1

Supplementary Table S4. Predictive performance for models based on questionnaire data only or administrative data only

Predictive performance for a positive test	Cut-off (%) for a positive test result			
	5	10	15	20
**Questionnaire**				
Detection rate	75.8	39.5	25.3	14.2
False positive rate	40.1	13.1	5.6	2.4
Ratio true to false positives	1 : 8.4	1 : 5.3	1 : 3.5	1 : 2.7
**Administrative data**				
Detection rate	73.7	34.7	20.0	8.4
False positive rate	45.2	12.4	4.3	1.7
Ratio true to false positives	1 : 9.7	1 : 5.7	1 : 3.4	1 : 3.2

Supplementary Table S5. Sensitivity, specificity and predictive value for models based on questionnaire only, administrative data only or predictors from both sources

Predictive performance for a positive test	Cut-off (%) for a positive test result			
	5	10	15	20
**Questionnaire**				
Sensitivity	75.8	39.5	25.3	14.2
Specificity	59.9	86.9	94.4	97.6
Positive predictive value	10.7	16.0	22.2	27.3
Negative predictive value	97.5	95.8	95.2	94.7
**Administrative data**				
Sensitivity	73.7	34.7	20.0	8.4
Specificity	54.8	87.6	95.7	98.3
Positive predictive value	9.3	15.0	22.8	23.9
Negative predictive value	97.1	95.5	95.0	94.4
**Questionnaire + administrative data**				
Sensitivity	75.3	51.1	34.2	26.8
Specificity	66.2	85.5	93.5	96.5
Positive predictive value	12.3	18.2	25.1	32.7
Negative predictive value	97.7	96.5	95.7	95.4

Supplementary Figure S1. Area Under Curve (AUC) for a 36-item model in relation to primary outcome (≥90-day sickness absence) and secondary outcomes (≥30-day sickness absence)

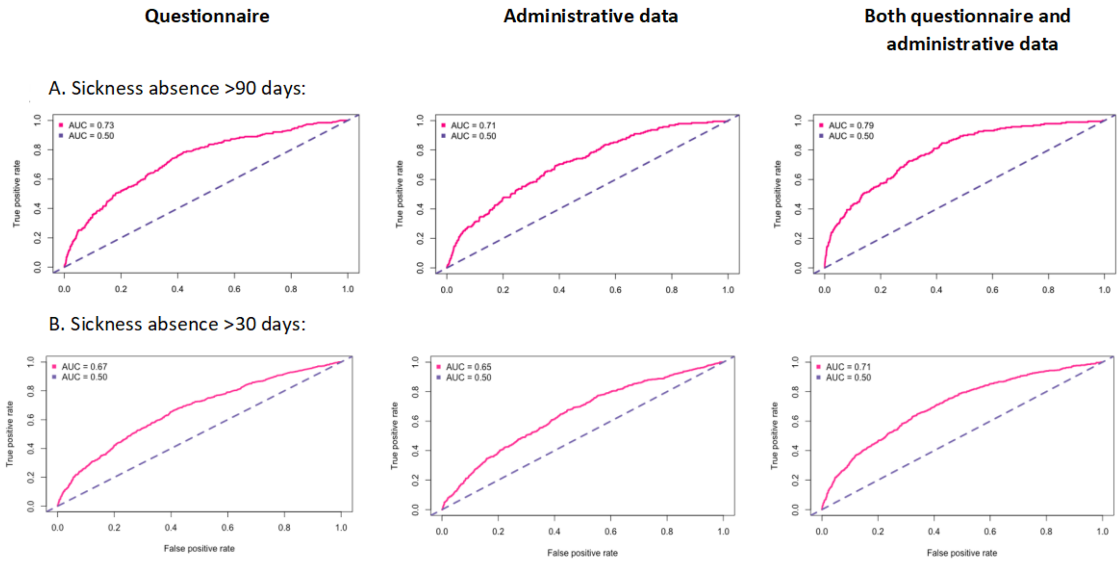

Supplementary Table S6. Predictive performance of a 36-item model in relation to primary outcome (≥90-day sickness absence) and secondary outcomes (≥30-day sickness absence)

Prediction metrics					
	Outcome: Sickness absence > 90 days				
Threshold for a positive test result	1	5	10	15	20
Detection rate, %	99.5	75.3	51.1	34.2	26.8
False positive rate, %	86.7	33.8	14.5	6.5	3.5
Ratio true to false positives	1 : 13.3	1 : 7.1	1 : 4.5	1 : 3.0	1 : 2.1
	Outcome: Sickness absence > 30 days				
Threshold for a positive test result	10	20	30	40	50
Detection rate, %	94.0	67.2	40.6	23.7	11.1
False positive rate, %	80.1	37.6	15.1	5.9	2.0
Ratio true to false positives	1 : 3.1	1 : 2.0	1 : 1.4	1 : 0.9	1 : 0.7

Supplementary Figure S2. Illustration of the capacity of the 36-item risk score to estimate risk of long-term sickness absence for primary and secondary outcomes

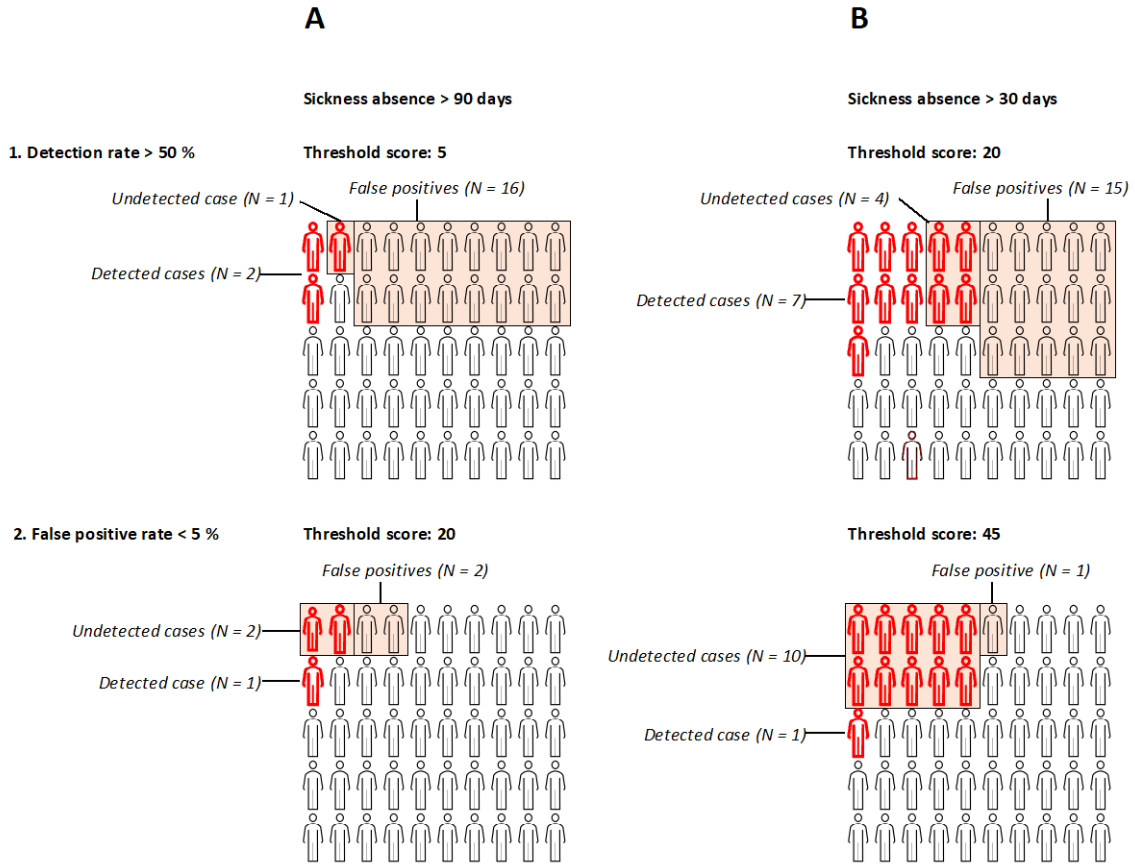

Supplementary Table S7. Variables selected by six approaches for feature selection (secondary outcome)

Predictor	Stepwise	Boruta	Lasso	Ridge	GA	Elastic net
**Corresponding AUC**	**0.69**	**0.7**	**0.7**	**0.7**	**0.69**	**0.68**
**Administrative data**						
Sex			x	x	x	
Long (>40 hour) working weeks	x	x	x		x	x
Long (>48 hour) working weeks		x		x	x	
Long shifts		x				
Long night shifts	x	x	x	x	x	
Early morning shifts						
Morning shifts		x	x	x		
Day shifts					x	
Evening shifts		x	x	x		
Night shifts		x	x	x		
Non-day shifts	x	x	x	x	x	x
Long spells of work shifts		x			x	
Short shift intervals		x	x	x		
Annual leave days			x	x	x	
Week-end work		x	x	x	x	
Single free days		x	x	x	x	
Realized shift plans		x	x			
Use of shift wishes	x	x	x	x	x	x
Number of staff				x	x	
Mean age of staff		x	x	x	x	
Proportion of employees aged 30 or younger		x		x	x	
Proportion of employees aged 60 or older	x		x	x	x	
Proportion of non-permanent staff		x		x		
Proportion of nurses	x	x	x	x	x	x
Low SES		x	x	x		x
Turnover (last 2 years)	x	x	x	x	x	x
Turnover (last 4 years)		x	x			
Rate of long sickness absence in the unit	x	x	x	x	x	x
**Questionnaire data**						
Age group	x		x	x	x	x
Body mass index (kg/m2) category			x	x		x
Socioeconomic status	x	x	x	x	x	
Smoking	x		x	x	x	x
No. of chronic diseases	x	x	x	x		x
Self-rated health	x	x	x	x	x	x
Trouble falling asleep		x	x	x	x	x
No. of sickness absences during the previous year	x	x	x	x	x	x

Supplementary Methods:

Statistical code

#model and AUC with all 36 potential predictor items (similar code used for models including items from one source only)

```
model3<-glm(sl90 ~ ., data=da4f,family="binomial")
summary(model3)
odds.n.ends(model3, rocPlot = TRUE)
```

#code for the stepwise-model

```
rr<-glm(sl90~ ., data=da2f,family="binomial")
step.model <- rr %>% stepAIC(trace = FALSE, method = "both")
coef(step.model)
summary(step.model)
terms2<- tidy(step.model)
terms2$term
```

#Machine learning models

```
#---------------- Boruta selection-----------------#
#Kursa, Miron B., and Witold R. Rudnicki. 2010. “Feature Selection with the Boruta Package.”
#Journal of Statistical Software 36 (11): 1–13. http://www.jstatsoft.org/v36/i11/.

jada2f <- da2f %>% drop_na()
set.seed(1)
library(Boruta)
boruta_output <- Boruta(sl90 ~ ., data=(jada2f), doTrace=2)

boruta_signif <- getSelectedAttributes(boruta_output, withTentative = TRUE)
print(boruta_signif)

roughFixMod <- TentativeRoughFix(boruta_output)
boruta_signif <- getSelectedAttributes(roughFixMod)
print(boruta_signif)

# Variable Importance Scores
imps <- attStats(roughFixMod)
imps2 = imps[imps$decision != 'Rejected', c('meanImp', 'decision')]
head(imps2[order(-imps2$meanImp), ])  # descending sort

plot(boruta_output, cex.axis=.7, las=2, xlab="", main="Variable Importance")
plot(boruta_output, cex.axis=.7, las=2, xlab="", main="Variable Importance (Boruta)")


# ----------- Lasso --------------#
```

```
jada2f <- da2f %>% drop_na()
x <- model.matrix(sl90~., jada2f)[,-1]
y <- as.numeric(jada2f$sl90)

set.seed(1)
cv.lasso <- cv.glmnet(x, y, alpha = 1, family = "binomial")
# Fit the final model on the training data
model <- glmnet(x, y, alpha = 1, family = "binomial",
            lambda = cv.lasso$lambda.min)
model2 <- glmnet(x, y, alpha = 1, family = "binomial",
             lambda = 0.01)
summary(model)
terms<- tidy(model)
terms$term
# Display regression coefficients
coef(model)

# ----------- Ridge --------------#

jada2f <- da2f %>% drop_na()
x <- model.matrix(sl90~., jada2f)[,-1]
y <- as.numeric(jada2f$sl90)

set.seed(1)
cv.lasso <- cv.glmnet(x, y, alpha = 0, family = "binomial")
# Fit the final model on the training data
model <- glmnet(x, y, alpha = 0, family = "binomial",
            lambda = cv.lasso$lambda.min)
model <- glmnet(x, y, alpha = 0, family = "binomial",
            lambda = cv.lasso$lambda.1se)
# model2 <- glmnet(x, y, alpha = 0, family = "binomial",
#              lambda = 0.01)
summary(model)
terms<- tidy(model)
terms$term
# terms2<- tidy(model2)
# terms2$term

ridge_coef = predict(model, type = "coefficients", s = cv.lasso$lambda.min)[1:96,] # Display
coefficients using lambda chosen by CV
ridge_coef
#ridge_coef[ridge_coef != 0]
ridge_coef[ridge_coef > 0.001]

#-------------------------- Genetic algorithm -------------------------------#
set.seed(27)
model_ga <- gafs(x = jada2f[, -1],
             y = as.factor(jada2f$sl90),
             iters = 10, # generations of algorithm
             popSize = 10, # population size for each generation
```

```
               levels = c("0", "1"),
               gafsControl = gafsControl(functions = rfGA, # Assess fitness with RF
                                         method = "cv",    # 10 fold cross validation
                                         genParallel = TRUE, # Use parallel programming
                                         allowParallel = TRUE))

model_ga$ga$final
model_ga$optVariables

#------------------- Elastic net --------------------------#
# Set training control
# train_cont <- trainControl(method = "repeatedcv",
#                          number = 10,
#                          repeats = 5,
#                          search = "random",
#                          verboseIter = TRUE)

elastic_reg <- caret::train(sl90 ~ .,
                       data = jada2f,
                       method = "glmnet",
                      # preProcess = c("center", "scale"),
                       tuneLength = 10,
                       trControl = trainControl(method = "repeatedcv",
                                                number = 10,
                                                repeats = 10,
                                                savePredictions = TRUE,
                                                verboseIter = FALSE))

# Best tuning parameter
elastic_reg$bestTune

importance <- varImp(elastic_reg, scale = TRUE)
plot(importance)
importance
```

Supplementary Table S8. STROBE Checklist

STROBE Statement—Checklist of items that should be included in reports of ***cohort studies***

	Item No	Recommendation
**Title and abstract**	1	(*a*) Indicate the study's design with a commonly used term in the title or the abstract **page 1**
		(*b*) Provide in the abstract an informative and balanced summary of what was done and what was found **pages 1-2**
**Introduction**		
Background/rationale	2	Explain the scientific background and rationale for the investigation being reported **page 3**
Objectives	3	State specific objectives, including any prespecified hypotheses **pages 4**
**Methods**		
Study design	4	Present key elements of study design early in the paper **page 4**
Setting	5	Describe the setting, locations, and relevant dates, including periods of recruitment, exposure, follow-up, and data collection **pages 4-6**
Participants	6	(*a*) Give the eligibility criteria, and the sources and methods of selection of participants. Describe methods of follow-up **pages 4-6**
Variables	7	Clearly define all outcomes, exposures, predictors, potential confounders, and effect modifiers. Give diagnostic criteria, if applicable **pages 4-6, Table 1, Table S1**
Data sources/ measurement	8*	For each variable of interest, give sources of data and details of methods of assessment (measurement). Describe comparability of assessment methods if there is more than one group **pages 4-6**
Bias	9	Describe any efforts to address potential sources of bias **pages 6-9**
Study size	10	Explain how the study size was arrived at **page 9, Figure 1**
Quantitative variables	11	Explain how quantitative variables were handled in the analyses. If applicable, describe which groupings were chosen and why **page 5-6, Table 1, Appendix Table S1**
Statistical methods	12	(*a*) Describe all statistical methods, including those used to control for confounding **pages 6-9**
		(*b*) Describe any methods used to examine subgroups and interactions
		(*c*) Explain how missing data were addressed **page 4-6**
		(*d*) If applicable, explain how loss to follow-up was addressed **NA**
		(*e*) Describe any sensitivity analyses **page 6**
**Results**		
Participants	13*	(a) Report numbers of individuals at each stage of study—eg numbers potentially eligible, examined for eligibility, confirmed eligible, included in the study, completing follow-up, and analysed **page 9, Figure 1**
		(b) Give reasons for non-participation at each stage
		(c) Consider use of a flow diagram **Figure 1**
Descriptive data	14*	(a) Give characteristics of study participants (eg demographic, clinical, social) and information on exposures and potential confounders **page 9, Table 1**
		(b) Indicate number of participants with missing data for each variable of interest **page 6**
		(c) Summarise follow-up time (eg, average and total amount) **page 9**
Outcome data	15*	Report numbers of outcome events or summary measures over time **page 9**
Main results	16	(*a*) Give unadjusted estimates and, if applicable, confounder-adjusted estimates and their precision (eg, 95% confidence interval). Make clear which confounders were adjusted for and why they were included **page 10-11, Figures 2-4**
		(*b*) Report category boundaries when continuous variables were categorized **page 5, Table 1, Table S1.**

		(*c*) If relevant, consider translating estimates of relative risk into absolute risk for a meaningful time period
Other analyses	17	Report other analyses done—eg analyses of subgroups and interactions, and sensitivity analyses **page 12**
**Discussion**		
Key results	18	Summarise key results with reference to study objectives **page 13**
Limitations	19	Discuss limitations of the study, taking into account sources of potential bias or imprecision. Discuss both direction and magnitude of any potential bias **pages 15-16**
Interpretation	20	Give a cautious overall interpretation of results considering objectives, limitations, multiplicity of analyses, results from similar studies, and other relevant evidence **pages 13-16**
Generalisability	21	Discuss the generalisability (external validity) of the study results **pages 16-17**
**Other information**		
Funding	22	Give the source of funding and the role of the funders for the present study and, if applicable, for the original study on which the present article is based **Title page**

*Give information separately for exposed and unexposed groups.

Note: An Explanation and Elaboration article discusses each checklist item and gives methodological background and published examples of transparent reporting. The STROBE checklist is best used in conjunction with this article (freely available on the Web sites of PLoS Medicine at http://www.plosmedicine.org/, Annals of Internal Medicine at http://www.annals.org/, and Epidemiology at http://www.epidem.com/). Information on the STROBE Initiative is available at http://www.strobe-statement.org.
